# Post-exercise rehydration: a randomized cross-over trial comparing a 100% fruit juice, a glucose-based sports drink, and water

**DOI:** 10.1080/15502783.2026.2721140

**Published:** 2026-08-31

**Authors:** Loris A. Juett, Jasper van der Wolf-Ong, Pierluigi A. Gyamfi, Shoheib D. Ebrahim, Emily B. Codd, Charles F. Pugh, Mark P. Funnell, John Hough, Ian Varley, David J. Clayton

**Affiliations:** a SHAPE Research Centre, School of Science and Technology, Nottingham Trent University, Nottingham, UK; b Leicester Diabetes Centre, Leicester General Hospital, University Hospitals of Leicester NHS Trust, Leicester, UK; c Diabetes Research Centre, College of Life Sciences, University of Leicester, Leicester, UK; d NIHR Applied Research Collaboration East Midlands, Diabetes Research Centre, University of Leicester, Leicester, UK

**Keywords:** Hypohydration, dehydration, water balance, glucose tolerance

## Abstract

**Background:**

Previous studies indicate that sports drinks may improve rehydration, compared to water, an effect likely achieved by manufacturing sports drinks to contain carbohydrates and sodium. However, there is a growing preference for natural products and a “food first” approach to sports nutrition. Fruit juices naturally contain similar concentrations of carbohydrates to sports drinks, but fruit juices may produce a more stable blood glucose profile. Fruit juices also naturally contain electrolytes, particularly potassium, but their potential as effective rehydration alternatives to sports drinks, which have higher sodium concentrations, is not well understood. This study compared the rehydration efficacy and glucose responses following consumption of a 100% fruit juice (Raw Hydrate®; FRU), a glucose-based sports drink (SPO), and water (WAT) after exercise-induced hypohydration. Importantly, rehydration beverages were matched for water volume, rather than total volume, to ensure that any potential differences in water balance were not due to unequal water volumes between trials, a limitation affecting previous rehydration research.

**Methods:**

After familiarization, 17 adults (age: 28 ± 8  years; BMI: 23.8 ± 2.9 kg/m^2^) completed three trials in a randomized cross-over design. The participants cycled in the heat (~35°C) to induce ~2% body mass loss (BML), then rehydrated over a 1 h period in a laboratory (~21°C) with a water volume equivalent to 150% of BML from either FRU, SPO, or WAT. This was followed by an additional 4 h of seated rest (5 h rehydration period), when blood glucose was measured (0, 0.25, 0.5, 0.75, 1, 1.5, and 2 h after beverage consumption), and all urine produced was collected.

**Results:**

During the 5 h rehydration period, there was no effect of trial on total urine volume (FRU: 1266 ± 403 mL, SPO: 1338 ± 361 mL, WAT: 1394 ± 360 mL; *P* = 0.156) or water retention (FRU: 42 ± 12%, SPO: 37 ± 10%, WAT: 35 ± 11%; *P* = 0.059). The blood glucose area under the curve differed by trial (*P* < 0.001), with all beverages significantly different from each other (FRU: 10.12 ± 0.72 mmol/L/2h, SPO: 12.48 ± 1.34 mmol/L/2h, WAT: 7.90 ± 0.47 mmol/L/2h; *P* < 0.003).

**Conclusion:**

Rehydration efficacy was similar between all beverages, but each elicited a distinct glycemic response. For sports drink consumers seeking a natural alternative or implementing a “food first” nutritional strategy, switching to a 100% fruit juice will not compromise rehydration effectiveness, but may elicit a lower blood glucose response. Although, it should be noted that an additional three participants were withdrawn from the study because of gastrointestinal issues after consuming the 100% fruit juice. This was likely a product of the present study’s design, where a large volume of 100% fruit juice (average ~2,300 mL) was consumed in a short period of time (1 h). Whilst this is a commonly used study design to robustly assess the rehydration efficacy of different beverages, future studies should distribute fluid intake over a longer duration, in order to improve ecological validity and reduce the risk of gastrointestinal issues.

## 1. Introduction

During exercise in temperate or hot conditions, the production and evaporation of sweat from the skin are important mechanisms for dissipating heat and attenuating increases in core body temperature. Under these conditions, sweat losses usually exceed fluid intake, resulting in hypohydration [1]. Emerging evidence suggests that hypohydration has the potential to accumulate over consecutive exercise sessions in regular exercisers [2], and athletes have been reported to begin exercise hypohydrated [3]. This is of concern because hypohydration may negatively affect exercise performance [4,5], cognitive performance [6], and mood [6], and may also increase the risk of adverse health outcomes, such as kidney stones [7] and urinary tract infections [8].

Following exercise-induced hypohydration, exercisers may opt for water as their rehydration beverage. However, the consumption of a large volume of plain water stimulates diuresis, resulting in low water retention and incomplete rehydration [9]. Previous studies have suggested that sports drinks may improve rehydration, compared to water, because of their carbohydrate (typically ~6%) and sodium (typically ~20 mmol/L) content [9–11]. Carbohydrates in sports drinks slow gastric emptying, moderating water absorption, which may attenuate disturbances to plasma volume and serum osmolality, whilst sodium may help maintain serum osmolality directly [10,12]. These combined effects likely explain the reduced urine production and greater water retention reported with sports drinks, compared to water [9–12]. However, a major challenge with interpreting previous rehydration literature is that drinks were typically matched for total volume, rather than for water volume. Due to the carbohydrate content of sports drinks, this would result in a lower volume of water being provided with a sports drink, compared to water. This additional water intake in the water trial may have increased diuresis, skewing rehydration variables (net fluid balance, urine volume, and drink retention) in favor of sports drinks. As such, it is unclear whether sports drinks improve rehydration, compared to water, when they are matched for water volume, rather than total volume.

Whilst sports drinks may improve rehydration, the regular consumption of large amounts of glucose and sodium (present in sports drinks) is associated with negative health outcomes. For example, high sodium intake can increase blood pressure [13], and the consumption of high-glucose drinks may cause hyperglycemia, which may acutely impair cognitive function and mood [14] and negatively affect metabolic health [15,16].

Compared to sports drinks, 100% fruit juices typically contain naturally lower concentrations of sodium and higher concentrations of potassium, an electrolyte that may improve rehydration [17], while also being associated with positive health outcomes, such as reduced blood pressure [18,19]. Furthermore, the types of carbohydrates provided in fruit juice may facilitate a more stable blood glucose profile [20,21]. Moreover, fruit juices are more closely aligned with a “food first” strategy, which is the preferred approach in sports nutrition because foods (such as fruit) provide energy and macronutrients alongside micronutrients, antioxidants, and phytochemicals, which can support health and performance [22,23]. However, the rehydration properties of fruit juices and their potential as effective alternatives to sports drinks, which have higher sodium concentrations, are not well understood.

The present study aimed to compare the rehydration efficacy and glucose responses following consumption of a 100% fruit juice (FRU), a glucose-based sports drink (SPO), and water (WAT), when matching beverages for their water volume (rather than total volume), after exercise-induced hypohydration. It was hypothesized that rehydration metrics (i.e. net water balance, urine volume, and water retention) would be similar for FRU and SPO, and that both FRU and SPO would rehydrate more effectively than WAT.

## 2. Materials and methods

### 2.1. Participants

The required sample size was estimated a priori, using G*Power 3.1 [24]. Using a medium effect size (partial η^2^ = 0.07; f = 0.27) for net water balance across three trials [25], an *α* of 0.05, a power of 0.8, and a correlation among repeated measures of 0.66 [25], the required sample size was estimated to be 17 participants. To account for attrition, twenty-nine participants were recruited for this study. Twelve participants were withdrawn from the study for the following reasons: unable to complete the exercise protocol (*n* = 3), diarrhea following consumption of 100% fruit juice (*n* = 3), non-compliance with study procedures (*n* = 3), scheduling conflicts (*n* = 2), and medication usage (*n* = 1) (Supplementary Figure 1). Seventeen healthy adults (15 males, 2 females; age: 28 ± 8 y; BMI: 23.8 ± 2.9 kg/m^2^; W_peak_: 238 ± 73 W), residing in the UK, completed this study.

Participants provided written informed consent before beginning the study, which was given a favorable opinion by the Nottingham Trent University Human Invasive Ethics Committee (01/10/2024; application ID: 1911625). This study was also retrospectively registered online (clinicaltrials.gov; NCT07468539). The participants completed a health screening questionnaire and were deemed healthy to participate in the study, including no history of kidney issues, liver issues, blood disorders, diabetes, or heat-related illness, and no regular medication usage. The participants were also screened for intolerances/allergies to all the foods and beverages provided in this study. The female participants were not pregnant, attempting to become pregnant, or breastfeeding. The menstrual cycle was not considered when scheduling trials, as previous studies indicate that gastric emptying [26] and rehydration [27] are not significantly affected by the menstrual cycle phase. Data collection took place in the UK from November 2024 to May 2025.

### 2.2. Study design

The participants visited the laboratory on four occasions: a screening and familiarisation trial, followed by three experimental trials in a randomized order with a cross-over design. Each experimental trial involved intermittent cycling in the heat (~35°C, ~50% relative humidity) to induce ~2% body mass loss (BML). Post-exercise, participants rehydrated in a temperate laboratory (~21°C) with either a 100% fruit juice (FRU), a glucose-based sports drink (SPO), or water (WAT). The water volume provided by each beverage was matched [28] to provide 150% of the participant’s BML, which was consumed over a 1 h period. The participants remained seated in the laboratory for an additional 4 h, completing a total 5 h rehydration period, during which blood glucose was measured, and all urine produced was collected. The experimental trials were separated by ≥7 days.

### 2.3. Beverages

The three rehydration beverages were: Raw Hydrate (cold-pressed watermelon [76%], pomegranate [20%], and lime [4%]; FRU; Innate-Essence Limited, London, UK), Lucozade Sport raspberry flavor (SPO; Lucozade Ribena Suntory Limited, Uxbridge, UK), and Highland Spring water (WAT; Highland Spring Limited, Perthshire, Scotland). The manufacturer reported values for macronutrients, salt (sodium chloride), and potassium content of each beverage are displayed in Table 1.

**Table 1.** Macronutrient, salt, and potassium contents of the trial beverages. Data are manufacturers' values.

Beverage	Raw hydrate (FRU)	Lucozade sport (SPO)	Highland spring water (WAT)
Carbohydrate (g/100 mL)	5.9	6.3	Negligible
Protein (g/100 mL)	1.0	Negligible	Negligible
Fat (g/100 mL)	Negligible	Negligible	Negligible
Salt (g/100 mL)	0.04	0.12	Negligible
Potassium (mg/100 mL)	135	Negligible	Negligible

In line with recent research [28], and given that our primary outcomes were net water balance, urine volume, and water retention, we applied a correction to the weights of FRU and SPO that were provided to participants, in order to account for the macronutrient content and ensure that the beverages delivered an equivalent volume of water. At the beginning of the study, 100 mL of three different FRU and SPO beverages were measured and weighed to the nearest 0.1 g (Kern PFB 6000-1, Balingen, Germany). The weight of macronutrients per 100  mL (Table 1) was then subtracted from the average weight of 100  mL of the beverages, allowing the conversion of beverage weights of FRU and SPO to the water content (FRU: 101.1 g beverage = 94.2 g water; SPO: 101.6 g beverage = 95.3 g water). As the macronutrient content of WAT was negligible, it was assumed that 1 g was equal to 1 mL of water.

### 2.4. Screening and familiarization trial

During the initial visit, participants had their height and body mass measured. Where necessary, participants were familiarized to fingertip capillary blood sampling and providing urine samples. The participants then completed a peak power output (W_peak_) test on a cycle ergometer (Lode, Groningen, The Netherlands) in a temperate environment (~21°C). The test began at 95 W and increased by 35 W every 3 min, until volitional exhaustion. After a self-determined rest period, the participants were familiarized with cycling in the heat (~35 °C, ~50% relative humidity). This involved completing two repeated blocks of cycling at ~50% W_peak_ for 10 min, each followed by 5 min of rest. The participants were also familiarized with the perceptual scales used during experimental trials, including the rating of perceived exertion (RPE; 6–20), thermal sensation (on a scale of -10 = unbearably cold to 10 = unbearably hot), and thirst (0–10 numbered scale; 0 = no symptom, 10 = maximum symptom). Additionally, they were familiarized with the measurements of aural temperature (Braun ThermoScan 3, Lausanne, Switzerland) and body mass (GFK-150H, Adam Equipment Co., UK).

### 2.5. Pre-trial standardisation

At 24 h before their first experimental trial, participants were instructed to avoid alcohol consumption and strenuous exercise, and to record their food and fluid (ensuring to consume  ≥40 mL/kg body mass fluid) intake using a food diary. The participants replicated these diet and exercise behaviors in the 24 h preceding their second and third experimental trials. Ninety minutes before arriving at the laboratory for the experimental trials, the participants finished a standardized breakfast of whole cereal bars (Nutri-Grain, Kellogg’s, Manchester, UK), providing 1–1.5 g carbohydrate/kg body mass, and 500 mL water. The participants received regular reminders to support adherence to pre-trial standardisation responsibilities, and compliance was confirmed verbally upon arrival for each experimental trial.

### 2.6. Experimental trial procedures

The participants arrived at the laboratory between 08:00 and 09:15 am (time standardized to within 30 min for each participant) and commenced seated rest, during which environmental temperature and humidity (TP49W digital indoor thermometer, ThermoPro, London, UK; used outside of the environmental chamber), thirst and GI comfort (0–10 numbered scale; 0 = no symptoms, 10 = maximum symptoms), and aural temperature (data not presented; only collected for safety purposes, due to exercise in the heat) were measured. After 10 min of rest, a blood sample (all blood samples taken were fingertip capillary blood samples) was taken before participants provided a urine sample (for all urine samples, participants fully emptied their bladder into a clean and dry container). At this timepoint, trials were discontinued if the urine osmolality exceeded 900 mOsm/kgH_2_O [5,29]. The participants then entered the environmental chamber (~35 °C, ~50% relative humidity), where body mass was measured (all body mass measurements were performed after the participants towel dried and weighed themselves in their underwear, behind a privacy screen).

The participants then commenced the dehydration period, which consisted of repeated blocks of 10  min cycling (~50% W_peak_) followed by 5  min of rest, during which body mass was measured. Eight minutes into each block of cycling, the environmental temperature and humidity (Kestrel, Nielsen-Kellerman Co., Boothwyn, USA; used inside the environmental chamber) were measured, followed by RPE, thermal sensation, thirst, and aural temperature. If aural temperature exceeded 38.5 °C, the trial was terminated and the participant was withdrawn from the study [30]. However, this threshold was not exceeded by any participant. If, during their first experimental trial, a participant showed difficulty completing a block of exercise, or if their aural temperature reached 38.4°C, the power output was reduced. This adjusted power output was then standardized for the participant's remaining experimental trials. Blocks of cycling and rest continued until participants reached ~1.5% BML, with the duration of the final block of cycling being adjusted accordingly. Once ~1.5% BML was achieved, participants exited the environmental chamber, with this time being recorded and defined as “time in the environmental chamber for dehydration”. The participants had 15  min to shower, with instructions to match the water temperature between trials, and return to the temperate laboratory for seated rest [17,25,31,32]. During this seated rest period, environmental temperature and humidity, thirst, GI comfort, and aural temperature were measured. Following 10 min seated rest, a blood sample was taken, urine was collected, and body mass was measured. It was anticipated that body mass would further decrease during this post-exercise period, resulting in a final BML of ~2%.

The participants then commenced the rehydration period, during which they consumed either FRU, SPO, or WAT. Each beverage in total provided an estimated water volume equivalent to 150% of the participant’s BML. This volume was divided into four equal aliquots that were provided to participants in opaque bottles every 15 min, with the drinking period lasting 1 h. One participant was unable to consume this volume within the allotted time during their first experimental trial and was therefore permitted to take longer (completed intake in 89 min). In this participant’s subsequent trials, this extended drinking period was replicated, but blood glucose was not measured. At the end of the drinking period, environmental temperature and humidity, thirst, and GI comfort were measured before a blood sample was taken and urine was collected. All of these measurements were taken hourly for the next 4 h (except blood samples, which were taken at 0.25, 0.5, 0.75, 1, 1.5, and 2 h after the drinking period), with participants resting in a seated position in the laboratory. If participants needed to urinate between scheduled hourly samples, they collected this urine, which was then pooled with the urine produced at the next scheduled hourly timepoint [17,28,31,32].

### 2.7. Sample analysis

All urine samples were weighed to the nearest 1 g before osmolality was measured using a handheld osmometer (Osmocheck, Vitech Scientific, Horsham, UK). It was assumed that 1 g = 1 mL, and thus urine volume was reported directly based on sample mass. Fingertip capillary blood samples were collected into 20 µL plastic capillary tubes, diluted 1:50 with haemolysing solution, and analysed for blood glucose concentrations using a benchtop analyser (Biosen C-Line, EKF diagnostics, HaB direct, Warwickshire, UK).

### 2.8. Calculations

1) Net water balance was calculated using the following formula:
([Post-exercisebodymass(kg)–pre-exercisebodymass(kg)]×1000)+watervolumeconsumed(g)–cumulativeurineoutputduringtherehydrationperiod(g).
2) Water retention was calculated using the following formula:
([watervolumeconsumed(g)–totalurinevolumeproducedduringtherehydrationperiod(g)]/watervolumeconsumed(g))×100.

### 2.9. Data and statistical analysis

As time in the environmental chamber varied between participants, measures taken during the dehydration period (environmental temperature and humidity, RPE, thermal sensation, and thirst) were averaged and compared between trials. Blood glucose data are presented for 16 participants, as we were unable to collect fingertip capillary blood samples from one participant (the same participant who required an extended rehydration period). Hypoglycemia was defined as <3.9 mmol/L, and hyperglycemia was defined as >7.8 mmol/L [33,34].

Statistical analyses were performed using SPSS Statistics version 29 (IBM, New York, USA). Data distribution were checked using a Shapiro–Wilk test. Data with one factor (trial) were analyzed using either a one-way repeated measures analysis of variance (ANOVA; parametric data) or the Friedman test (non-parametric data). Data with two factors (trial and time) were analyzed using a two-way repeated measures ANOVA. If the data violated the assumption of sphericity, the Greenhouse–Geisser correction was used. Significant effects of trial (data with one and two factors) and trial-by-time interaction effects (data with two factors) were followed up by post hoc paired *t*-tests (parametric data) or Wilcoxon signed-ranks tests (non-parametric data). To reduce the risk of type 1 errors, the Bonferroni correction was applied to post hoc tests. Data are presented as mean ± standard deviation, and statistical significance was defined as *P* ≤ 0.05.

## 3. Results

### 3.1. Baseline measures and trial conditions

**Figure 1.**
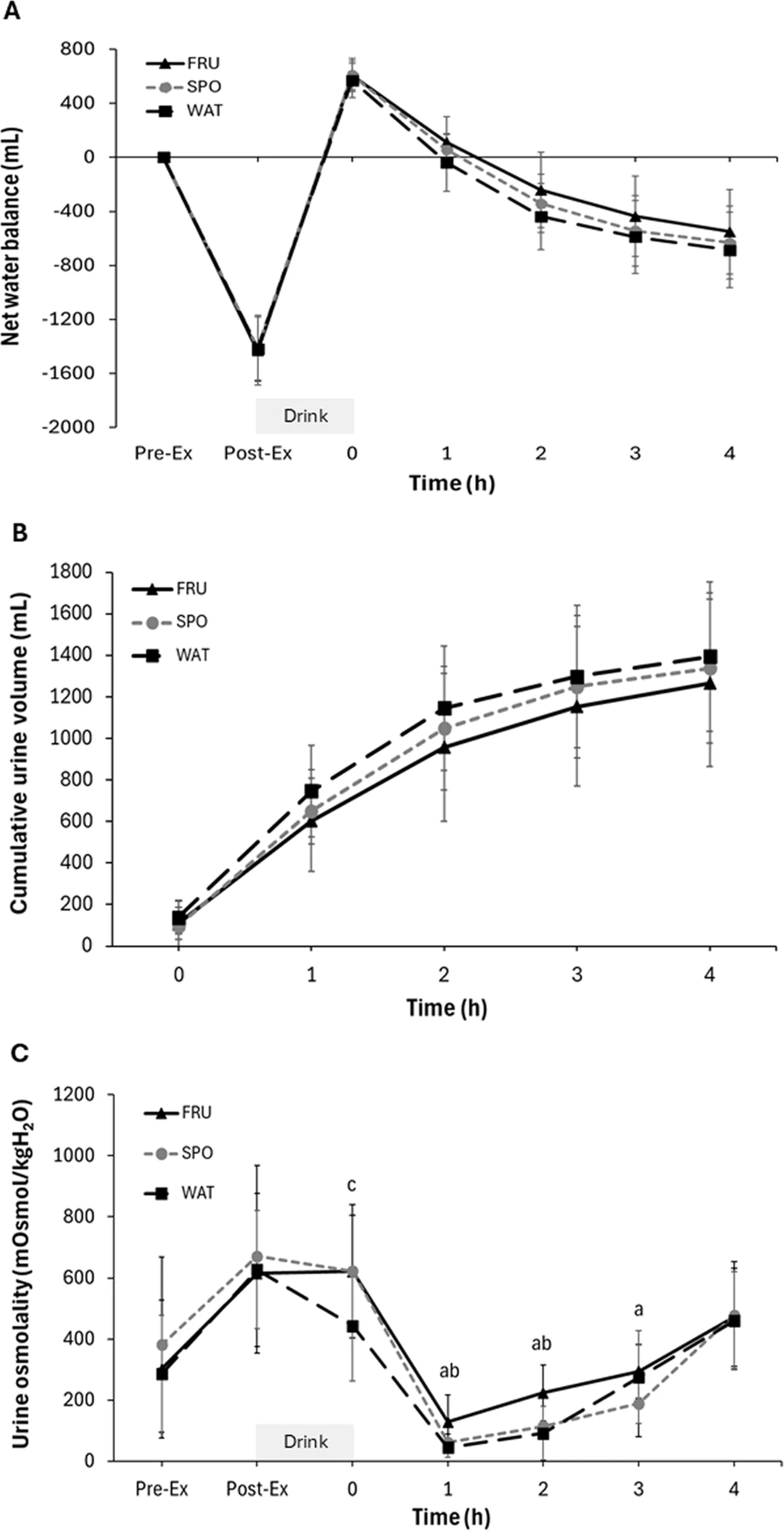
(A) Net water balance, (B) cumulative urine volume, and (C) urine osmolality pre-exercise (Pre-Ex), post-exercise (Post-Ex), and 0, 1, 2, 3, and 4 h following rehydration (after drinking period) with either a 100% fruit beverage (FRU), glucose-based sports drink (SPO), or water (WAT). a = FRU significantly different to SPO, b = FRU significantly different to WAT, c = SPO significantly different to WAT.

**Figure 2.**
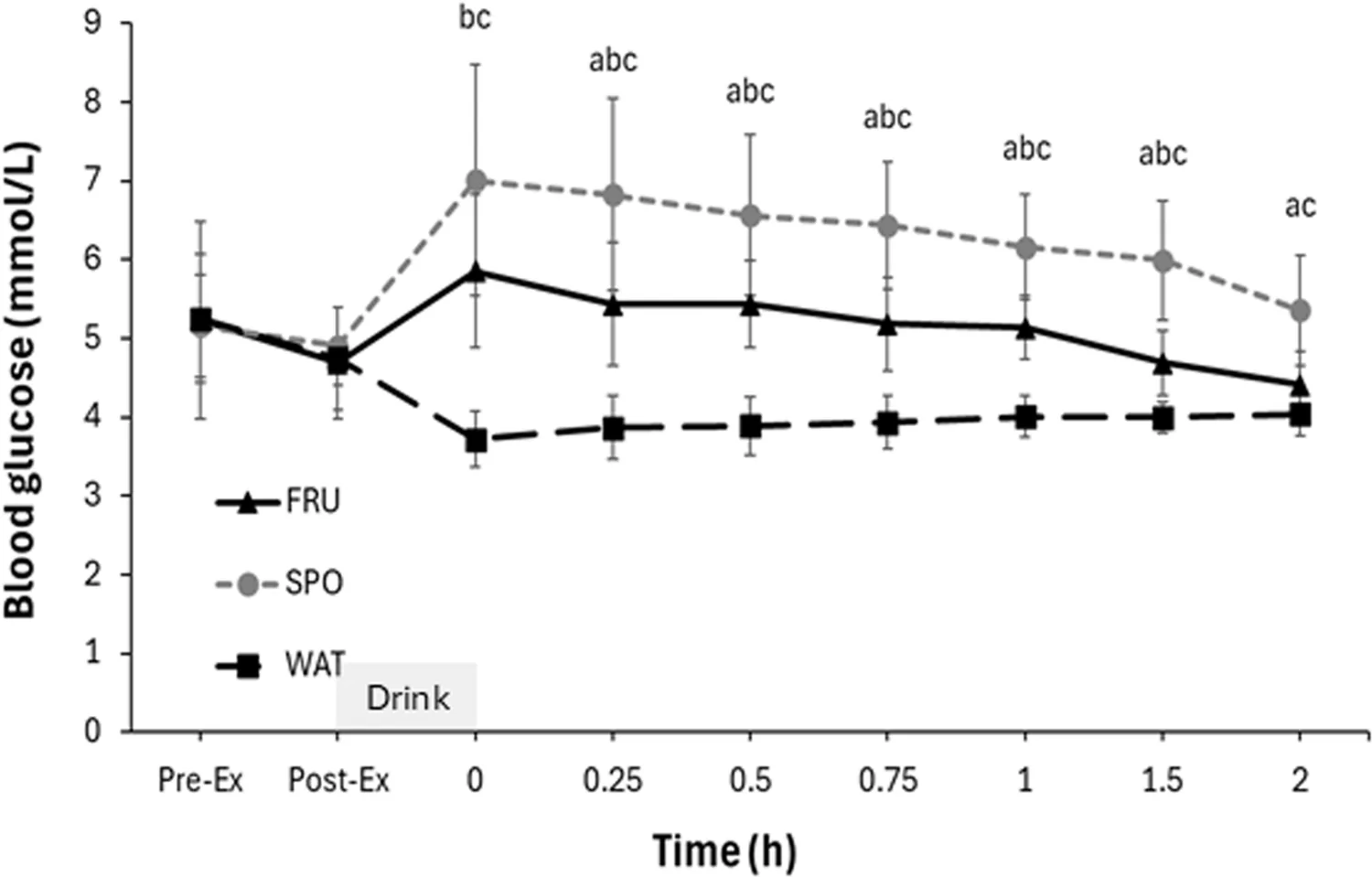
Blood glucose concentrations pre-exercise (Pre-Ex), post-exercise (Post-Ex), and 0, 0.25, 0.5, 0.75, 1, 1.5, and 2 h following rehydration (after the drinking period) with either a 100% fruit beverage (FRU), a glucose-based sports drink (SPO), or water (WAT). a = FRU significantly different to SPO, b = FRU significantly different to WAT, c = SPO significantly different to WAT.

**Figure 3.**
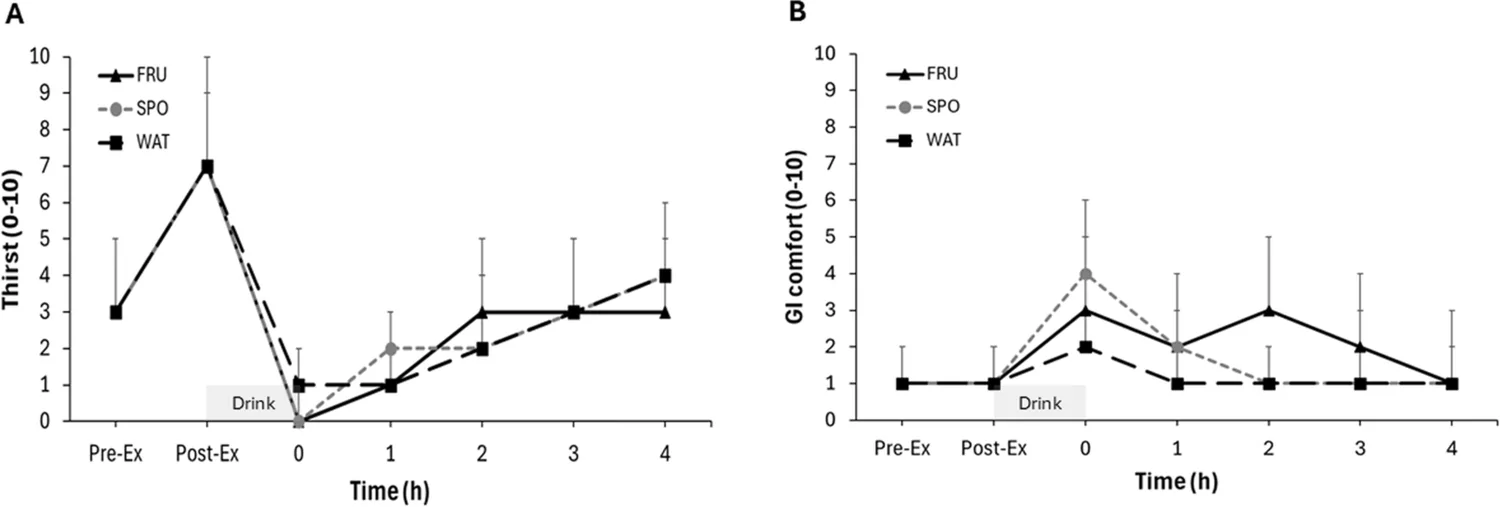
(A) Thirst perception (0–10) and (B) gastrointestinal (GI) comfort (0–10) at pre-exercise (Pre-Ex), post-exercise (Post-Ex), and 0, 1, 2, 3, and 4 h following rehydration (after drinking period) with either a 100% fruit beverage (FRU), glucose-based sports drink (SPO), or water (WAT).

At baseline, there was no effect of trial on body mass (FRU: 74.43 ± 10.84 kg, SPO: 74.21 ± 11.06 kg, WAT: 74.56 ± 10.86 kg; *P* = 0.106), urine osmolality (*P* = 0.096; Figure 1C), blood glucose concentrations (*P* = 0.646; Figure 2), thirst (*P* = 0.298; Figure 3A) or GI comfort (*P* = 0.882; Figure 3B), indicating that participants arrived in a similar physiological state for trials, having followed pre-trial standardisation.

There was no effect of trial on laboratory temperature (FRU: 21.3 ± 1.1 °C, SPO: 21.2 ± 1.1 °C, WAT: 20.9 ± 1.2 °C; *P* = 0.419) and humidity (FRU: 35 ± 5%, SPO: 35 ± 4%, WAT: 34 ± 4%; *P* = 0.827) or environmental chamber temperature (FRU: 34.8 ± 0.4 °C, SPO: 34.8 ± 0.3 °C, WAT: 34.8 ± 0.4 °C; *P* = 0.852) and humidity (FRU: 55.4 ± 3.0%, SPO: 55.2 ± 3.4%. WAT: 55.1 ± 3.6%; *P* = 0.390).

### 3.2. Dehydration period

There was no effect of trial (*P* ≥ 0.308) on RPE, thermal sensation, thirst, time in the environmental chamber for dehydration, absolute body mass loss, or relative body mass loss during the dehydration period (Table 2), indicating that the severity of dehydration and physiological and perceptual responses during the dehydration period were consistent across all experimental trials.

**Table 2.** Physiological and perceptual data collected during the dehydration period for the three experimental trials.

	FRU	SPO	WAT	*p*–value
RPE	13 ± 2	13 ± 2	13 ± 2	0.595
Thermal sensation	4 ± 1	4 ± 1	4 ± 2	0.975
Thirst	6 ± 2	5 ± 2	5 ± 2	0.492
Time in environmental chamber for dehydration (min)	90 ± 14	91 ± 17	91 ± 14	0.544
Body mass loss (g)	1432 ± 251	1413 ± 243	1416 ± 233	0.580
Body mass loss (%)	1.92 ± 0.15	1.90 ± 0.14	1.90 ± 0.13	0.308

FRU = 100% fruit beverage; SPO = glucose-based sports drink; WAT = water; RPE = rating of perceived exertion.

### 3.3. Water balance and urine osmolality

There was no effect of trial (*P* = 0.580) on the water volume provided by each beverage (FRU: 2148 ± 377 mL, SPO: 2119 ± 365 mL, WAT: 2125 ± 349 mL). However, there was an effect of trial (*P* < 0.001) on total beverage weight provided, with both FRU (2305 ± 405 g) and SPO (2260 ± 389 g) significantly greater (*P* ≤ 0.003) than WAT (2125 ± 349 g), but there was no difference between FRU and SPO (*P* = 0.177).

There was an effect of trial (*P* = 0.035) on net water balance (Figure 1A), but post hoc tests revealed no significant differences (*P* ≥ 0.129). There was a trial-by-time interaction effect (*P* = 0.034) for net water balance. However, post hoc tests revealed no significant differences between trials at any timepoint (*P* ≥ 0.36). There was no effect of trial (*P* = 0.054) or trial-by-time interaction effect (*P* = 0.099) for cumulative urine volume during the rehydration period (Figure 1B). There was a trial-by-time interaction effect (*P* = 0.003) for urine osmolality (Figure 1C). There was no effect of trial on the total urine volume produced during the rehydration period (FRU: 1266 ± 403 mL, SPO: 1338 ± 361 mL, WAT: 1394 ± 360 mL; *P* = 0.156) or percentage of water retained (water retention) from the beverages at the end of the rehydration period (FRU: 42 ± 12%, SPO: 37 ± 10%, WAT: 35 ± 11%; *P* = 0.059). Water retention was highest in the FRU for 10 participants, in the SPO for 3 participants, and in the WAT for 3 participants, with one participant tied (to one decimal place) between FRU and WAT.

### 3.4. Blood glucose

There was a trial-by-time interaction effect (*P* < 0.001) for blood glucose concentrations (Figure 2), with all trials significantly different from each other (*P* < 0.027) at 0.25, 0.5, 0.75, 1, and 1.5 h following finishing the beverages. Immediately following finishing the beverages, blood glucose concentrations were greater in FRU compared to WAT (*P* < 0.027), and in the SPO compared to WAT (*P* < 0.027). Two hours following finishing the beverages, blood glucose concentrations were greater in SPO compared to FRU (*P* < 0.027), and in SPO compared to WAT (*P* < 0.027). During the rehydration period, hyperglycemia (blood glucose > 7.8 mmol/L) was observed in 7 participants (44%) in SPO, but none (0%) in FRU or WAT, and hypoglycemia (blood glucose < 3.9 mmol/L) was detected in 12 participants (75%) in WAT, 3 (19%) in FRU, and none (0%) in SPO. There was an effect of trial (*P* < 0.001) on the blood glucose area under the curve (AUC) following finishing the beverages, with all trials significantly different from each other (FRU: 10.12 ± 0.72 mmol/L/2 h, SPO: 12.48 ± 1.34 mmol/L/2 h, WAT: 7.90 ± 0.47 mmol/L/2 h; *P* < 0.003). For all participants, the blood glucose AUC was greatest in SPO, followed by FRU, followed by WAT, indicating a consistent pattern of response.

### 3.5. Perceptual scales

There was no effect of trial (*P* = 0.420) or trial-by-time interaction effect (*P* = 0.128) for thirst (Figure 3A). There was an effect of trial (*P* = 0.028) on GI comfort (Figure 3B), but post hoc tests revealed no significant differences (*P* ≥ 0.087). There was a trial-by-time interaction effect (*P* = 0.002) for GI comfort. However, post hoc tests revealed no significant differences between trials at any timepoint (*P* ≥ 0.147).

## 4. Discussion

The main findings from the present study were: (1) net water balance, urine volume, and water retention did not differ between beverages, indicating that a 100% fruit juice (FRU), glucose-based sports drink (SPO), and water (WAT) had similar rehydration efficacies; and (2) FRU, SPO, and WAT all significantly differed in their blood glucose responses, with FRU producing lower blood glucose concentrations than SPO. These findings suggest that 100% fruit juices achieve a similar degree of rehydration to a sports drink, but elicit a lower blood glucose response.

These results support the use of a 100% fruit juice for effective post-exercise rehydration, comparable to commonly consumed sports drinks and water. However, the present study also found no difference in rehydration between water and a sports drink, which contrasts previous studies [9–11]. These contrasting findings may be due to the present study matching beverages for water volume, rather than total volume. In line with previous research [28], we applied a correction factor to the weights of FRU and SPO to account for macronutrient content and match water intake between trials. Whilst we acknowledge that we were unable to correct for all the dissolved constituents, and therefore some uncertainty remains regarding the exact water content of the beverages, macronutrients account for the majority of the dissolved constituents. Therefore, any error introduced by other dissolved constituents was likely small and unlikely to meaningfully influence the main finding that FRU, SPO, and WAT had similar rehydration efficacies. Previous studies typically matched drink volume, rather than water volume, but this confounds comparisons of rehydration efficacy, owing to unequal amounts of water consumed between trials. For example, a 500 mL 6% glucose sports drink contains at least 30 mL less water than a matched volume of 500 mL plain water (possibly more, depending on other ingredients), with this disparity increasing as drink volume increases. Consequently, a reduction in urine volume produced after consuming the sports drink may reflect, at least in part, the lower water volume ingested, rather than an improvement in water retention attributable to drink content.

Given the lack of significant differences between beverages for rehydration variables in the present study, it seems that higher concentrations and amounts of carbohydrates (FRU: ~5.9%; SPO: ~6.3%), sodium chloride (SPO: 2.7 ± 0.5 g, ~20 mmol/L; FRU: 0.9 ± 0.2 g, ~7 mmol/L), or potassium (FRU: 3.1 ± 0.5 g, ~35 mmol/L) would have been required to significantly improve rehydration, when water intake is matched. Owing to previously described methodological differences, it is difficult to determine the carbohydrate concentration required to improve rehydration. Whilst concentrations ≥ 10% seem to be required to improve water retention [31,35,36], a high carbohydrate concentration would have created a significant disparity in water intake between trials in these prior studies. Consequently, the effects of carbohydrate on rehydration are not fully understood. In contrast to carbohydrate, when manipulating sodium/potassium content, the difference between matching drink volume versus water volume is likely negligible. Therefore, when considering the findings from the present study alongside previous research [32,37], it can be broadly concluded that a sodium concentration of ≥ 40 mmol/L is likely required to significantly improve rehydration. With regards to potassium, the concentration required to significantly improve rehydration is less clear, with concentrations of 30  mmol/L [9] and ~35 mmol/L (present study) having been shown not to significantly improve rehydration. However, in contrast, a concentration of 25 mmol/L potassium chloride has been shown to significantly improve rehydration [17]. Although these studies differ in terms of overall beverage composition, these findings raise the possibility that factors beyond potassium concentration alone, such as the anion accompanying potassium, may influence rehydration efficacy.

Whilst we identified trends for an effect of trial on cumulative urine volume (*P* = 0.054) and water retention (*P* = 0.059) in the present study, these trends disappeared when Bonferroni-corrected post hoc tests were applied (*P* ≥ 0.162). In addition, the mean differences in cumulative urine volume between beverages (56–128 mL/5 h) were small and unlikely to be of practical significance. Previous studies examining the addition of 40–50 mmol/L sodium chloride have reported substantially larger average differences in cumulative urine volume (approximately 400–700 mL) between beverages at a similar time point [32,37].

It was anticipated that WAT would produce the lowest blood glucose response, but it was notable that 12 participants experienced hypoglycemia after consuming WAT, compared to 3 participants after consuming FRU and 0 after consuming SPO. The blood glucose response was significantly lower after consuming FRU compared to SPO, despite their similar carbohydrate contents (5.9 g/100 mL and 6.3 g/100 mL, respectively). This suggests that carbohydrate source and composition are important considerations, rather than solely total carbohydrate quantity. SPO contains glucose (from glucose syrup), whereas the carbohydrate in FRU comprised of glucose, fructose, and sucrose (from cold-pressed watermelon, pomegranate, and lime). The carbohydrate contents of watermelons and pomegranates (the primary constituents of FRU) vary, but we would estimate that the carbohydrate from watermelon would have been ~30% glucose, ~40% fructose, and ~30% sucrose [21], whereas the carbohydrate from pomegranate would have been ~54% fructose and ~43% glucose [20]. Our findings indicate that 100% fruit juices lead to more stable blood glucose concentrations than glucose-based sports drinks and water.

Whilst chronically high intakes of fructose could have negative effects on cardiometabolic health, it seems that the evidence to support this theory in humans derives from studies that have given very high amounts of fructose-sweetened beverages (not fruit juices), which resulted in a calorie surplus and weight gain [38–40]. Therefore, these findings are unlikely to apply to the context of rehydration with 100% fruit juices following exercise, when reductions in liver glycogen likely mitigate the effects of fructose ingestion on de novo lipogenesis [41,42]. The 100% fruit juice used in the present study (Raw Hydrate; 76% watermelon, 20% pomegranate, and 4% lime) would also contain an array of phytochemicals and antioxidants, including vitamin A, vitamin C, lycopene, L-citrulline, anthocyanins, and ellagitannins, which may support health and provide performance benefits, such as reduced inflammation and muscle soreness [43–45].

Whilst there were no significant differences between beverages for gastrointestinal comfort scores among the participants who completed the present study, these findings should be interpreted cautiously because the analysis did not include the data from the three participants who were withdrawn from the study due to experiencing diarrhea following FRU consumption. All three of these participants experienced diarrhea during the laboratory visit, within 2 h following the 1 h drinking period, and reported that their diarrhea and gastrointestinal discomfort had settled later that day. No other participants reported experiencing diarrhea either during any of their laboratory visits or after they had left the laboratory. The exact cause of this diarrhea is not known, but it may have been due to a high fructose load. This was likely a product of the present study’s design, where a large volume of 100% fruit juice (average ~2300 mL), and therefore large amounts of carbohydrates (~136 g) and fructose (~68 g, assuming ~50% fructose), were provided in a short period of time (1 h). For some individuals, this may have exceeded the small intestine’s ability to absorb fructose, resulting in unabsorbed fructose reaching the distal small intestine or proximal large intestine, drawing in water and accelerating gut transit time, resulting in diarrhea [46]. Spreading this volume of fluid out over a longer period of time, as would typically be done in a real-world setting, would have likely significantly reduced the risk of this occurring.

The present study provided large volumes of fluid to be consumed in a short period of time, in line with most of the past rehydration literature [12]. Whilst this is a commonly used study design that robustly assesses the rehydration characteristics of different drinks in a controlled setting, this approach lacks ecological validity. Though there are instances where an athlete may not consume food post-exercise, due to a lack of food availability and/or appetite [47], it is unlikely that an athlete would consume such a large volume of fluid within 1 h and then refrain from eating or drinking for the following 4 h. Therefore, future studies should consider distributing fluid intake over a longer duration, and incorporating food intake, to improve ecological validity and reduce the risk of adverse gastrointestinal events. They should also consider measuring specific GI symptoms, rather than solely measuring GI comfort.

The present study has several limitations. Firstly, the equations used for net water balance and water retention in the present study and previous rehydration studies [25,31,32,36,37] did not account for other ongoing fluid losses, such as sweat loss or insensible losses, in the rehydration period. Consequently, these equations may have slightly overestimated net water balance and water retention. However, given that the present study used a cross-over design and that laboratory temperature and humidity did not significantly differ between trials, any unmeasured fluid losses were likely similar between trials and therefore unlikely to have meaningfully influenced the main findings. Secondly, there was an imbalance in participant sex (15 males, 2 females), meaning that the findings primarily reflect responses in males and cannot confidently be generalized to females. Thirdly, participants and researchers were not blinded to the experimental beverages. Efforts were made to minimize bias, with participants not informed of all beverages being tested (they were informed that the study compared two sports/hydration drinks with water), two similarly flavored experimental beverages selected, and all drinks provided in opaque containers. While lack of blinding represents a limitation, it is unlikely to have influenced the primary objective outcome measures (net water balance, urine volume, and water retention).

In conclusion, the findings from the present study provide novel evidence that a 100% fruit juice provided post-exercise rehydration comparable to that of a commonly consumed glucose-based sports drink and water. For sports drink consumers, a 100% fruit juice is a similarly effective rehydration alternative that produces a lower blood glucose response.

## Supplementary Material

Supplementary Material Supplementary_material.docx

## Data Availability

The data that support the findings of this study are available from the corresponding author upon reasonable request.

## References

[1] Cheuvront SN , Haymes EM . Thermoregulation and marathon running: biological and environmental influences. Sports Med. 2001;31(10):743–762. doi: 10.2165/00007256-200131100-00004 11547895

[2] Funnell MP , Juett LA , Ferrara R , et al. Ad-libitum fluid intake was insufficient to achieve euhydration 20 h after intermittent running in Male team sports athletes. Physiol Behav. 2023;268:114227. doi: 10.1016/j.physbeh.2023.114227 37156317

[3] Volpe SL , Poule KA , Bland EG . Estimation of prepractice hydration status of national collegiate athletic association division I athletes. J Athl Train. 2009;44(6):624–629. doi: 10.4085/1062-6050-44.6.624 19911089 PMC2775364

[4] Funnell MP , Embleton D , Morris T , et al. Exercise-induced hypohydration impairs 3 km treadmill-running performance in temperate conditions. J Sports Sci. 2023;41(12):1171–1178. doi: 10.1080/02640414.2023.2259728 37733070

[5] Funnell MP , Mears SA , Bergin-Taylor K , et al. Blinded and unblinded hypohydration similarly impair cycling time trial performance in the heat in trained cyclists. J Appl Physiol (1985). 2019;126(4):870–879. doi: 10.1152/japplphysiol.01026.2018 30629476

[6] Ganio MS , Armstrong Le , Casa DJ , et al. Mild dehydration impairs cognitive performance and mood of men. Br J Nutr. 2011;106(10):1535–1543. doi: 10.1017/S0007114511002005 21736786

[7] Fink HA , Akornor JW , Garimella PS , et al. Diet, fluid, or supplements for secondary prevention of nephrolithiasis: a systematic review and meta-analysis of randomized trials. Eur Urol. 2009;56(1):72–80. doi: 10.1016/j.eururo.2009.03.031 19321253 PMC2925677

[8] Hooton TM , Vecchio M , Iroz A , et al. Effect of increased daily water intake in premenopausal women with recurrent urinary tract infections: a randomized clinical trial. JAMA Intern Med. 2018;178(11):1509–1515. doi: 10.1001/jamainternmed.2018.4204 30285042 PMC6584323

[9] Shirreffs SM , Aragon-Vargas LF , Keil M , et al. Rehydration after exercise in the heat: a comparison of 4 commonly used drinks. Int J Sport Nutr Exerc Metab. 2007;17(3):244–258. doi: 10.1123/ijsnem.17.3.244 17693686

[10] González-Alonso J , Heaps CL , Coyle EF . Rehydration after exercise with common beverages and water. Int J Sports Med. 1992;13(5):399–406. doi: 10.1055/s-2007-1021288 1521958

[11] Ly NQ , Hamstra-Wright KL , Horswill CA . Post-exercise rehydration in athletes: effects of sodium and carbohydrate in commercial hydration beverages. Nutrients. 2023;15(22):4759. doi: 10.3390/nu15224759 38004153 PMC10674530

[12] Evans GH , James LJ , Shirreffs SM , et al. Optimizing the restoration and maintenance of fluid balance after exercise-induced dehydration. J Appl Physiol. 2017;122(4):945–951. doi: 10.1152/japplphysiol.00745.2016 28126906

[13] Huang L , Trieu K , Yoshimura S , et al. Effect of dose and duration of reduction in dietary sodium on blood pressure levels: systematic review and meta-analysis of randomised trials. BMJ. 2020;368:m315. doi: 10.1136/bmj.m315 32094151 PMC7190039

[14] Sommerfield AJ , Deary IJ , Frier BM . Acute hyperglycemia alters mood state and impairs cognitive performance in people with type 2 diabetes. Diabetes Care. 2004;27(10):2335–2340. doi: 10.2337/diacare.27.10.2335 15451897

[15] Ceriello A , Esposito K , Piconi L , et al. Oscillating glucose is more deleterious to endothelial function and oxidative stress than mean glucose in normal and type 2 diabetic patients. Diabetes. 2008;57(5):1349–1354. doi: 10.2337/db08-0063 18299315

[16] Merovci A , Tripathy D , Chen X , et al. Effect of mild physiologic hyperglycemia on insulin secretion, insulin clearance, and insulin sensitivity in healthy glucose-tolerant subjects. Diabetes. 2021;70(1):204–213. doi: 10.2337/db20-0039 33033064 PMC7881846

[17] Maughan RJ , Owen JH , Shirreffs SM , et al. Post-exercise rehydration in man: effects of electrolyte addition to ingested fluids. Eur J Appl Physiol Occup Physiol. 1994;69(3):209–215. doi: 10.1007/BF01094790 8001531

[18] Geleijnse JM , Kok FJ , Grobbee DE . Blood pressure response to changes in sodium and potassium intake: a metaregression analysis of randomised trials. J Hum Hypertens. 2003;17(7):471–480. doi: 10.1038/sj.jhh.1001575 12821954

[19] Greer RC , Marklund M , Anderson CAM , et al. Potassium-enriched salt substitutes as a means to lower blood pressure: benefits and risks. Hypertension. 2020;75(2):266–274. doi: 10.1161/HYPERTENSIONAHA.119.13241 31838902

[20] Hasnaoui N , Jbir R , Mars M , et al. Organic acids, sugars, and anthocyanins contents in juices of Tunisian pomegranate fruits. Int J Food Prop. 2011;14(4):741–757. doi: 10.1080/10942910903383438

[21] Yativ M , Harary I , Wolf S . Sucrose accumulation in watermelon fruits: genetic variation and biochemical analysis. J Plant Physiol. 2010;167(8):589–596. doi: 10.1016/j.jplph.2009.11.009 20036442

[22] Close GL , Kasper AM , Walsh NP , et al. Food first but not always food Only": recommendations for using dietary supplements in sport. Int J Sport Nutr Exerc Metab. 2022;32(5):371–386. doi: 10.1123/ijsnem.2021-0335 35279015

[23] Reynolds KM , Juett LA , Cobb J , et al. Apple puree as a natural fructose source provides an effective alternative to artificial fructose sources for fuelling endurance cycling performance in males. Nutraceuticals. 2022;2(3):205–217. doi: 10.3390/nutraceuticals2030015

[24] Faul F , Erdfelder E , Lang A , et al. G*Power 3: a flexible statistical power analysis program for the social, behavioral, and biomedical sciences. Behav Res Methods. 2007;39(2):175–191. 17695343 10.3758/bf03193146

[25] Peden DL , Funnell MP , Reynolds KM , et al. Post-exercise rehydration: comparing the efficacy of three commercial oral rehydration solutions. Front Sports Act Living. 2023;5:1158167. doi: 10.3389/fspor.2023.1158167 37181252 PMC10174327

[26] Caballero-Plasencia AM , Valenzuela-Barranco M , Martín-Ruiz JL , et al. Are there changes in gastric emptying during the menstrual cycle? Scand J Gastroenterol. 1999;34(8):772–776. 10499477 10.1080/003655299750025697

[27] Rodriguez-Giustiniani P , Galloway SDR . Influence of peak menstrual cycle hormonal changes on restoration of fluid balance after induced dehydration. Int J Sport Nutr Exerc Metab. 2019;29(6):651–657. doi: 10.1123/ijsnem.2019-0105 31629351

[28] Peden DL , Derbyshire S , Funnell MP , et al. Fluid and electrolyte balance following consumption of skimmed milk and a plant-based soya beverage at rest in euhydrated males. Eur J Appl Physiol. 2024;124(10):3085–3093. doi: 10.1007/s00421-024-05516-0 38809478 PMC11467101

[29] Armstrong LE , Pumerantz AC , Fiala KA , et al. Human hydration indices: acute and longitudinal reference values. Int J Sport Nutr Exerc Metab. 2010;20(2):145–153. doi: 10.1123/ijsnem.20.2.145 20479488

[30] Juett LA , Drury JE , Greensmith TB , et al. Hypohydration induced by prolonged cycling in the heat increases biomarkers of renal injury in males. Eur J Appl Physiol. 2024;124(4):1085–1096. doi: 10.1007/s00421-023-05328-8 37848571 PMC10954877

[31] Clayton DJ , Evans GH , James LJ . Effect of drink carbohydrate content on postexercise gastric emptying, rehydration, and the calculation of net fluid balance. Int J Sport Nutr Exerc Metab. 2014;24(1):79–89. doi: 10.1123/ijsnem.2013-0024 23980237

[32] Maughan RJ , Leiper JB . Sodium intake and post-exercise rehydration in man. Eur J Appl Physiol Occup Physiol. 1995;71(4):311–319. doi: 10.1007/BF00240410 8549573

[33] De Meulemeester J , Charleer S , Visser MM , et al. The association of chronic complications with time in tight range and time in range in people with type 1 diabetes: a retrospective cross-sectional real-world study. Diabetologia. 2024;67(8):1527–1535. doi: 10.1007/s00125-024-06171-y 38787436

[34] Jarvis PRE , Cardin JL , Nisevich-Bede PM , et al. Continuous glucose monitoring in a healthy population: understanding the post-prandial glycemic response in individuals without diabetes mellitus. Metabolism. 2023;146:155640. doi: 10.1016/j.metabol.2023.155640 37356796

[35] Osterberg KL , Pallardy SE , Johnson RJ , et al. Carbohydrate exerts a mild influence on fluid retention following exercise-induced dehydration. J Appl Physiol. 2010;108(2):245–250. doi: 10.1152/japplphysiol.91275.2008 19940093

[36] Evans GH , Shirreffs SM , Maughan RJ . Postexercise rehydration in man: the effects of osmolality and carbohydrate content of ingested drinks. Nutrition. 2009;25(9):905–913. doi: 10.1016/j.nut.2008.12.014 19487107

[37] Merson SJ , Maughan RJ , Shirreffs SM . Rehydration with drinks differing in sodium concentration and recovery from moderate exercise-induced hypohydration in man. Eur J Appl Physiol. 2008;103(5):585–594. doi: 10.1007/s00421-008-0748-0 18463891

[38] Lê K , Ith M , Kreis R , et al. Fructose overconsumption causes dyslipidemia and ectopic lipid deposition in healthy subjects with and without a family history of type 2 diabetes. AJCN. 2009;89(6):1760–1765. 10.3945/ajcn.2008.27336 19403641

[39] Stanhope KL , Schwarz JM , Keim NL , et al. Consuming fructose-sweetened, not glucose-sweetened, beverages increases visceral adiposity and lipids and decreases insulin sensitivity in overweight/obese humans. J Clin Invest. 2009;119(5):1322–1334. doi: 10.1172/JCI37385 19381015 PMC2673878

[40] Rizkalla SW . Health implications of fructose consumption: a review of recent data. Nutr Metab (Lond). 2010;7:82. doi: 10.1186/1743-7075-7-82 21050460 PMC2991323

[41] Hyson DA . A review and critical analysis of the scientific literature related to 100% fruit juice and human health. Adv Nutr. 2015;6(1):37–51. doi: 10.3945/an.114.005728 25593142 PMC4288278

[42] Hengist A , Koumanov F , Gonzalez JT . Fructose and metabolic health: governed by hepatic glycogen status? J Physiol. 2019;597(14):3573–3585. doi: 10.1113/JP277767 30950506 PMC6767689

[43] Basu A , Penugonda K . Pomegranate juice: a heart-healthy fruit juice. Nutr Rev. 2009;67(1):49–56. doi: 10.1111/j.1753-4887.2008.00133.x 19146506

[44] Manivannan A , Lee E , Han K , et al. Versatile nutraceutical potentials of Watermelon-A modest fruit loaded with pharmaceutically valuable phytochemicals. Molecules. 2020;25(22):5258. doi: 10.3390/molecules25225258 33187365 PMC7698065

[45] Vitošević B , Filipović M , Popović L , et al. Juice-based supplementation strategies for athletic performance and recovery: a systematic review. Sports (Basel). 2025;13(8):269. doi: 10.3390/sports13080269 40863778 PMC12389966

[46] Gibson PR , Newnham E , Barrett JS , et al. Review article: fructose malabsorption and the bigger picture. Aliment Pharmacol Ther. 2007;25(4):349–363. doi: 10.1111/j.1365-2036.2006.03186.x 17217453

[47] Thackray AE , Stensel DJ . The impact of acute exercise on appetite control: current insights and future perspectives. Appetite. 2023;186:106557. doi: 10.1016/j.appet.2023.106557 37044176

