# Supplementary material for: Post-exercise rehydration: a randomized cross-over trial comparing a 100% fruit juice, a glucose-based sports drink, and water
Source: J Int Soc Sports Nutr. 2026 Aug 31;23(1):2721140. doi: 10.1080/15502783.2026.2721140 (PMC13532359; doi:10.1080/15502783.2026.2721140)
Supplement: Supplementary file 1 [file RSSN_A_2721140_SM5733.docx]

Supplementary Fig.1: CONSORT flow diagram.
